# The Application of Preventive Medicine in the Future Digital Health Era

**DOI:** 10.2196/59165

**Published:** 2025-02-27

**Authors:** Katherine De la Torre, Sukhong Min, Hyobin Lee, Daehee Kang

**Affiliations:** 1 Department of Biomedical Sciences Seoul National University Graduate School Seoul Republic of Korea; 2 Department of Preventive Medicine Seoul National University College of Medicine Seoul Republic of Korea; 3 Integrated Major in Innovative Medical Science Seoul National University Graduate School Seoul Republic of Korea

**Keywords:** preventive medicine, personalized prevention, digital health technology, digital health, artificial intelligence, wearable devices, telemedicine

## Abstract

A number of seismic shifts are expected to reshape the future of medicine. The global population is rapidly aging, significantly impacting the global disease burden. Medicine is undergoing a paradigm shift, defining and diagnosing diseases at earlier stages and shifting the health care focus from treating diseases to preventing them. The application and purview of digital medicine are expected to broaden significantly. Furthermore, the COVID-19 pandemic has further accelerated the shift toward predictive, preventive, personalized, and participatory (P4) medicine, and has identified health care accessibility, affordability, and patient empowerment as core values in the future digital health era. This “left shift” toward preventive care is anticipated to redefine health care, emphasizing health promotion over disease treatment. In the future, the traditional triad of preventive medicine—primary, secondary, and tertiary prevention—will be realized with technologies such as genomics, artificial intelligence, bioengineering and wearable devices, and telemedicine. Breast cancer and diabetes serve as case studies to demonstrate how these technologies such as personalized risk assessment, artificial intelligence–assisted and app-based technologies, have been developed and commercialized to provide personalized preventive care, identifying those at a higher risk and providing instructions and interventions for healthier lifestyles and improved quality of life. Overall, preventive medicine and the use of advanced technology will hold great potential for improving health care outcomes in the future.

## Introduction

As populations age and chronic diseases become more prevalent, societies will face challenges in providing equitable health care. Currently, a shift in the medical paradigm is ongoing, with more focus on keeping people healthier for longer and preventing diseases rather than just curing them. This approach acknowledges the importance of maintaining, improving, and promoting health throughout the natural progression of diseases and the increasing importance of preventive medicine.

In this new paradigm, cutting-edge technologies such as big data, informatics, and medical digitalization will allow for personalized and precise health care recommendations based on a patient’s clinical, biological, and genomic information. In addition, the availability of health-related information and the use of wearable devices have further encouraged individuals to take proactive measures in preventing health problems. The COVID-19 pandemic has also highlighted the importance of telemedicine in remote patient monitoring and providing more equitable health care.

Overall, preventive medicine and the use of advanced technology will hold great potential for improving health care outcomes in the future. However, the implementation of such technologies presents challenges such as addressing disparities in technology access, managing overuse and overreliance on digital tools, safeguarding patient data, and ensuring clinical validity, which call for further research in this area.

This review aims to elucidate the role of preventive medicine through the integration of digital health technologies and provide insights for the changing landscape of preventive health care in the digital era. By examining current trends, technological advancements, and case studies, we will explore how these innovations can lead to a more proactive, personalized, and patient-centered approach to health promotion and disease prevention.

## Future Trend and Emerging New Technologies

### Rise in Older Adult Population

The global population is aging, with people worldwide living longer lives. Nowadays, most individuals are expected to reach their 60s and beyond, a trend that is prevalent in every country. By the year 2030, approximately 1 out of every 6 people around the world is expected to be 60 years or older. The number of individuals in this age group is projected to increase from 1 billion in 2020 to 1.4 billion by 2030, and to double to 2.1 billion by 2050. Moreover, the number of individuals aged 80 years or older is anticipated to triple, reaching 426 million by 2050. Some countries such as Korea and Japan have been labeled as “super-aged societies” due to their pronounced aging trend, with 30% or more of their population being older than 65 years by 2050 [1].

This change in population composition is expected to have far-reaching health implications. The most striking is the increased burden of chronic diseases [2]. One study has projected that by 2035, 36%, and by 2050, 48% of adults older than 50 years will have at least 1 chronic disease [3]. As the global population ages and the burden of chronic diseases rises, medical costs have also increased worldwide due to longer care duration and health-related productivity loss. This has led to an annual growth of US $4 in the average Organization for Economic Cooperation and Development health expenditure per capita over the past decade [4]. In the case of the United States, 1 study has estimated that approximately 20% of health care spending growth will be attributable to aging by 2025 [5].

### Shift From Cure to Prevention

There is currently a general shift of health care away from merely providing cure to that of prevention, as the definition of health transitions from “the absence of disease” to “realizing the full potential of one’s capacity.” The paradigm change in medicine has been attributed to the humanization movement in medicine, which has contributed to changes in medical education, patient care, and the management of several medical conditions [6]. Nowadays, there is a steady transition from “cure-seeking medical care” to “cure and support-seeking medical care” [7], which has necessitated the development of new models of conceptualizing behavior change, emphasizing assessment of multiple risks, and treating the whole patient to optimize health outcomes [8,9].

The recent change in medical paradigms has impacted various aspects of health toward a more patient-centered approach, emphasizing patient engagement, health maintenance, and the restoration of daily activity. Specifically, where the health care of the past was episodic, focused on service providers and individual patients at independent and isolated institutions, health care is expected to increasingly shift toward a continuous care model. In this model, the general public and the community as a whole come to prevent diseases and maintain their health, and, if the need for treatment or hospitalization arises, are closely followed through after their discharge [10]. The patients are also expected to be better informed, with health care workers and patients sharing a more equal footing (Table 1) [10,11].

**Table 1:** Current and future health care.

Categories	Current	Future
Goal	Disease treatment	Health promotion
Continuity of care	Episodic cure Fragmented care	Continuing care Comprehensive care
Focus of care	Focus on service provider Individual approach	Focus on well-informed patient Team-based approach
Care setting	Hospital-centered care	Patient-/home-oriented care
Payment system	Pay for procedures	Pay for value

### Usage of Digital Health Technology

The convergence of life sciences and engineering disciplines has ushered in an era of medical technology integration, where genomics, artificial intelligence (AI), bioengineering, wearable devices, and telemedicine are used for various medical applications (Table 2). Advancements in technology have greatly impacted the medical field, with the development of bionic eye devices or 3D bioprinting technology used in various medical fields or wearable devices such as Google Glass have been used in surgical settings, offering benefits such as improved surgical education, training, consultation, patient monitoring, and audiovisual recording, among others [12].

**Table 2:** New technologies used for future medicine.

Domain	Description	Application
Genomics	Study of an individual’s entire genome. Revealing inherited traits and susceptibility to diseases.	Whole-genome sequencing for personalized medicine. Identification of genetic markers associated with diseases.
Artificial intelligence	Mimic human intelligence based on algorithms and computational models. Application in big data analysis, image recognition, and problem-solving.	Machine and deep learning algorithms for medical image analysis. Predictive models for disease diagnosis and prognosis.
Biomedical devices	Integration of engineering principles with biological systems. Design and development of devices to enhance medical treatments.	Artificial organs and tissue engineering for transplantation. Biomedical implants for drug delivery or physiological monitoring.
Wearable devices	Collect real-time data related to health and activities. Enhance continuous monitoring and personalized health care.	Smartwatches monitoring sleep, ECG^a^, and stress patterns. Medical wearables for continuous glucose monitoring or ECG recording.
Telemedicine	Delivery of health care services remotely. Comprises video consultations, remote monitoring, and virtual care.	Video calls for remote doctor consultations. Remote patient monitoring for disease management.

^a^ECG: electrocardiograms.

The expansion and use of digital health is evident in daily life through mobile health applications for wellness, nutrition, medication adherence, fitness tracking, and access to personal health information. Health information technology includes electronic medical record systems and cloud storage, which improve the accessibility, sharing, and accuracy of patient data. Electronic prescriptions and communication between doctors and patients are also improved. Wearable devices, wireless sensors, and diagnostic products play a role in monitoring health and diagnostics, such as continuous blood pressure, glucose, or sleep patterns monitoring.

Hence, a key goal of digital technology in health care is digital therapeutics, which uses evidence-based therapeutic interventions software integrated with mobile devices, apps, sensors, and virtual reality to prevent, manage, and treat diseases or disorders. These can be used alone or in combination with mechanical devices, pharmacological treatments, or in-person therapy, having a positive impact because they are designed to cater to the specific needs of patients. Therefore, the customization of health services through digital technology is essential for personalized health care, including patient-reported outcomes, predictive analytics, and clinical decision support.

Advancements in internet of things sensors and health tech wearables have made it easier to collect and transmit health data. The decreasing cost of internet of things sensors, along with the improved capabilities of consumer-grade devices such as the Apple Watch, has made it more affordable to equip individuals with tools to monitor their health. For example, the Apple Watch’s electrocardiogram functionality can detect irregular heartbeats and even send notifications for conditions such as atrial fibrillation. Other wearable devices, such as smart continuous glucose monitors and connected inhalers, offer benefits such as insulin-level monitoring and medication adherence. In addition, researchers are exploring the use of smartphones for “digital phenotyping” to identify individuals at risk of mental health problems [13,14]. With increased data access and sharing, these devices have the potential to provide highly personalized care based on individual patient profiles.

As digital technology continues to advance, personalized prevention and patient engagement in health monitoring and treatment management will become more feasible. Telemedicine and telehealth have become increasingly popular, especially for non–face-to-face virtual visits, remote patient monitoring, and remote care programs, which have been particularly relevant during the COVID-19 pandemic. The future of health care is heavily reliant on digital health, particularly for preventive medicine, as health information becomes more accessible to everyone and patient health empowerment increases.

### Changes in Health Care After COVID-19

Telemedicine was previously used in limited cases where access to in-person health care was difficult. However, the COVID-19 pandemic highlighted its importance in addressing health care labor shortages, reducing the risk of new infections in clinics, and providing care to vulnerable populations. As a result, telemedicine usage significantly increased during the pandemic and has a more widely discussed and used tool in health care [15]. This accelerated adoption of telemedicine now includes various medical specialties, including preventive medicine.

The future of health care in the post–COVID-19 era relies on 3 key aspects: accessibility, affordability, and empowerment (Table 3) [16]. Telemedicine has greatly improved accessibility by bringing care and remote monitoring closer to patients, regardless of their location. It has also facilitated rapid diagnoses and disease treatments, reduced costs, and prepared health care systems for future pandemic situations. For example, a report on the adoption of telehealth in Australia during the COVID-19 pandemic noted that for every in-person consultation replaced with telemedicine, up to 2.5 days of travel time and US $215 in societal productivity were saved [17]. Similar findings were echoed in the United States [18] and Canada [19].

**Table 3:** Key aspects of the future of health care after COVID-19.

Key aspects and implementations		Effects
**Accessibility**		
	Telemedicine	Bring care closer to patients Remote monitoring
	Point-of-care diagnostics	Rapid diagnosis and treatment Reduced cost Quick response for future pandemic
**Affordability**		
	Cost-effective medical devices	Innovative, low cost, and easy-to-use portable devices
	Self-health management	Empowered health seekers Lower the cost by avoiding hospitalization
**Empowerment**		
	Personalized health records	Support mass screening and epidemiological programs Automatization for an efficient health care ecosystem
	Patient engagement and education	Health awareness with education Disease awareness with wearables

As medical technology continues to advance, more affordable and innovative devices are becoming available in the market. There is a growing emphasis on developing portable medical devices that are cost-effective, easy to use, and accessible to everyone. The popularity of digital technology and smart wearables is steadily increasing, as they complement clinical care and provide valuable health data, such as vital signs, sleep patterns, and physical activity levels. These data can help predict disease progression and enable a shift toward preventive health care, empowering patients to actively manage their own health and reduce health care costs and limit health facilities use.

Furthermore, empowering patients in health care in the future involves leveraging electronic health records and integrated medical and digital devices to support mass screening and epidemiological programs. It also involves automating supply chains, where blockchain technology may be a vital asset, to improve efficiency across the health care ecosystem. Patient education and health-disease awareness are crucial components of the health care ecosystem and can be facilitated through web-based health content platforms and the use of smart wearables.

The advancement of medical technology, coupled with the impact of telemedicine following the COVID-19 pandemic and the shift in the medical paradigm, is set to transform the health care industry and the future of medicine. This transformation will result in a shift toward predictive, preventive, personalized, and participatory (P4) medicine, with a focus on individualized patient profiling and tailored prevention strategies. This new paradigm emphasizes patient-centered care, aims to improve overall health outcomes, and will highlight even more the importance of preventive medicine in the future of health care [20,21].

### Left Shift of the Natural Progression of Disease

The field of medicine has evolved since the age of epidemics from the age of complex diseases to the current age of postgenomics [22]. In the age of epidemics, the control of pathogens was the main treatment approach highlighting the importance of public hygiene. In the age of complex diseases, disease was based on multifactorial living conditions and lifestyle factors and care was empirically derived from trial and error experience of prior practice or observations. During this era, the distinction between treatment and prevention in the field of medicine became clearer. The growth of genetics research and completion of the Human Genome Project have introduced the concept of genetic susceptibility and gene-environment interaction to the age of postgenomics. In this era, the development of personalized medicine accelerated and the definition of health and disease began to shift. The new disease paradigm is a shift in the natural progression from treatment to health promotion and prevention.

**Figure 1:**
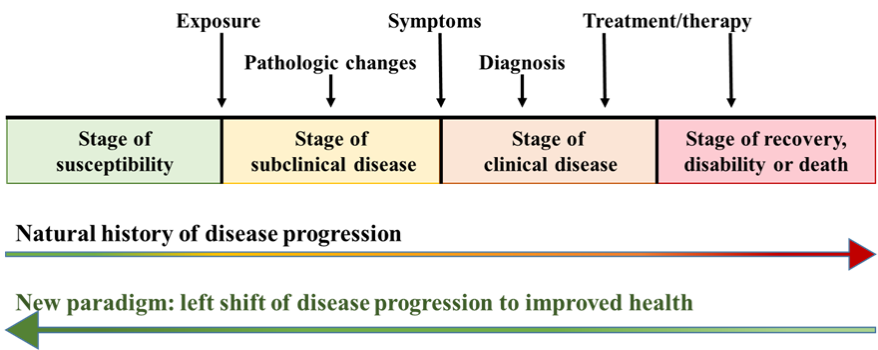
Left shift of natural history of disease progression.

A few decades ago, the main goal of medical practitioners was to accurately diagnose and treat diseases. The focus was on the clinical stage, with the aim of finding the best treatment, ensuring quick recovery, and preventing disease recurrence. However, the medical care paradigm has since shifted to the left (Figure 1) to focus on the stage before clinical symptoms appear, where there are subclinical manifestations and pathological changes. Medical care at this stage, that is, early detection, involves identifying pathological or structural changes that occur before symptoms manifest over the progression of the natural history of the disease. The discovery and integration of biomarkers have played a significant role in this approach. However, even at an earlier stage, some people are more likely than others to be affected by carcinogens or substances; therefore, understanding individual variations in susceptibility to risk factors has become crucial for disease prevention.

This shift in the paradigm of medical care is also reflected in our understanding and diagnosis of diseases. For instance, autism spectrum disorder (ASD), one of the most common neurodevelopmental disorders, has been diagnosed and named differently in previous versions of the *DSM* (*Diagnostic and Statistical Manual of Mental Disorders*) published by the American Psychiatric Association [23]. In earlier *DSM* versions, modern-day ASD was considered schizophrenia occurring before puberty. From *DSM-III* (*Diagnostic and Statistical Manual of Mental Disorders* [Third Edition]) to *DSM-IV-TR* (*Diagnostic and Statistical Manual of Mental Disorders* [Fourth Edition, Text Revision]), autism was distinguished from schizophrenia with the introduction of pervasive developmental disorder under which specific diagnoses expanded from 3 to 5. Eventually, the term “spectrum” and clinical characteristic specifiers were adopted to capture the wide range of symptoms and severity in ASD (*DSM-V* [*Diagnostic and Statistical Manual of Mental Disorders* {Fifth Edition}]). This highlights the importance of a diagnostic classification that can tailor interventions and care to individual needs [24]. In the future, we will see diseases on a continuous scale, and treatment will be personalized to each case. The focus of future medicine will be on providing continuous care rather than a one-time single type of care.

Overall, the prevailing paradigm for natural disease progression is steadily shifting toward health promotion and prevention, making preventive medicine, early diagnosis, and early detection, strategies that aim to reduce the burden of mortality and morbidity from diseases, essential. The value of disease prevention has been highlighted by evidence that suggests that prevention is more valuable than treatment. In the future, the paradigm of medical care is expected to increasingly shift from the stage of treating illness, disability, or death toward promoting overall health and disease prevention.

## Future of Preventive Medicine

### Current and Future Preventive Medicine

The American Board of Preventive Medicine defines preventive medicine as “the specialty of medical practice that focuses on the health of individuals, communities, and defined populations” and cites protection, promotion, and maintenance of health and well-being, and prevention of disease, disability, and death as some of the field’s core goals. In practice, these goals are achieved through 3 distinct types of prevention. Primary prevention aims to prevent the development of diseases in healthy individuals. It includes measures such as smoking cessation programs, vaccinations, and reducing alcohol consumption. Secondary prevention focuses on early detection of diseases to minimize their severity and complications. Examples include cancer-screening programs, tumor biomarkers, and diet or exercise programs to prevent further cardiovascular disease complications. Tertiary prevention aims to reduce the impact of existing diseases or ongoing injuries. It involves prompt treatment and additional approaches such as rehabilitation to prevent complications and minimize lasting effects.

While the conventional prevention triad’s approach is still valid, preventive medicine needs technological updates to enable personalized prevention strategies and better disease management, focusing on individual and public health needs. In fact, technology plays a crucial role in the current focus on disease prevention (Table 4). For one, the future of primary prevention care lies in the implementation of disease risk prediction services that use genomic information, such as polygenetic risk scores for cancer risk. 23andMe, for one, provides genetic health screening, offering “Health Predisposition Reports” that screen for genetic risk factors for diabetes; breast, ovarian, prostate, and pancreatic cancer; and chronic kidney disease, among many others, allowing formation of informed health plans and healthy life choices.

Personalized coaching services to reduce weight, control diet, or shape individual training programs will also play a significant role. Digital health care can overcome the limitations of traditional health care services by creating tailored services that are easy, affordable, efficient, and shaped according to individual needs. KakaoHealthcare Pasta, for example, provides app-based diet management service specialized in diabetes prevention, analyzing which foods most influence blood glucose levels and providing dietary suggestions accordingly. The current and future use of devices will also allow continuous monitoring of body vitals and biomarkers such as glucose, heart rate, and blood pressure. Collected information can be used to keep track of the user’s health trend, healthy and unhealthy activities, and provide guides to lead a healthy lifestyle. Noom is one such a personalized health record app that records diet, physical activity, and mood, and provides healthier recipes, encourages and reminds its users to exercise, and offers psychological tools for lasting health improvement.

**Table 4:** Classical and digital-based preventive medicine based on prevention types.

Prevention type	Classical	Digital-based
Primary	Smoking/alcohol abstinence advice Unstructured exercise recommendations Generic dietary recommendations	Genetic health risk screening Personalized health record apps App-based diet management
Secondary	Human operator–dependent cancer screenings Demographic-based cancer screening routines Annual health checkups	AI^a^-enhanced medical screening Personalized cancer screening Continuous monitoring
Tertiary	In-person rehabilitation clinics Standardized cancer chemotherapy Intermittent blood glucose testing	Telerehabilitation Gene-based prognosis/treatment Continuous blood glucose monitoring

^a^AI: artificial intelligence.

Digital technology could aid secondary prevention, detecting diseases in their early, asymptomatic stages and allowing for timely intervention before symptoms develop. This proactive approach aims to identify risk factors and prevent the progression of the disease and to detect noncommunicable disease complications early. Artificial intelligence, specifically machine learning (ML), has made significant advancements in using large amounts of patient data to enhance decision-making and predictions. ML is already being used in clinical settings to support health care professionals in tasks such as diagnosing respiratory issues from computed tomographic scans [25,26] or assisting with triage decisions through chatbots [27].

ML can also identify patterns and indicators that predict which patients will require more intensive medical interventions in the future [28]. ML also helps make better therapeutic decisions by pinpointing the treatments with the highest chances of success for each patient and their specific condition [29].

One example is Vuno, which provides AI-assisted medical imaging interpretations and diagnoses in a wide range of applications from simple chest radiographs to identifying signs of cardiac arrests and degenerative brain diseases. Chronic diseases such as diabetes and hypertension are recognized as conditions that exist on a continuous spectrum between health and disease. By using serum biomarkers and regular clinical checkups, people spend years in borderline states such as “prediabetes” or “prehypertension.” Today’s technology can help ascertain these conditions at these early stages, allowing for preventive measures.

In addition, advancements in genomics have allowed for the identification of genetic susceptibility to diseases such as breast cancer. This knowledge enables individuals to take proactive measures to prevent the disease. HexaMed is one such solution that collects anthropometric measurements and lifestyle choices in a simple questionnaire to predict cancer risk and suggests genomic, imaging, biomarker screening, and lifestyle changes.

Medical technology will also help shape individual rehabilitation for tertiary prevention. Individual dose calculations and drug delivery strategies based on each patient’s characteristics and diseases, including risk factors; genetic, genomic, and biomarker information; and social, behavioral, and economic conditions, will drive individualized treatment approaches. OncoFree, for example, provides breast cancer prognosis prediction using next-generation sequencing, and in addition, its results can be used to form postsurgical plans by providing clinicians with an estimate of how effective chemotherapy will be.

### Case Study 1: Breast Cancer

Breast cancer is the most common cancer and cause of cancer mortality globally [30]. Its rising prevalence is in part driven by lifestyle factors such as poor diet and inactivity [31,32]. Although mammography screening has enhanced early detection and improved prognosis, in many countries, the screening method is recommended through a one-size-fits-all approach. However, the absolute risk for breast cancer for the majority of the population is lower than average, whereas a small portion is at a significantly higher risk. Identifying who is at a higher risk and providing a more personalized screening based on each individual’s risk factors can optimize health care resources allocation, ensuring appropriate preventive interventions for high-risk individuals and enhancing overall breast cancer prevention efforts [33].

Technological advancements in digital health could significantly enhance the prevention, early detection, and treatment of breast cancer—that is, primary, secondary, and tertiary prevention (Table 5). For example, telehealth and telemedicine, actively used for monitoring and follow-up of patients with cancer during and after COVID-19 [34], have been shown to be effective for primary and secondary breast cancer prevention. In aiding patients with *BRCA* mutations, while the level of satisfaction toward counseling for cancer-specific psychological distress was found to be comparable whether the counseling was delivered in-person or remotely, the participants perceived remotely delivered genetic counseling as more convenient and less expensive [35].

**Table 5:** Prevention strategies and commercial applications according to the level of prevention.

Prevention type	Prevention strategies
Primary	General advice for a healthy lifestyle. Mammography screening frequency tailored to risk.
Secondary	Discuss preventive therapies. Individual counseling.
Tertiary	Preventive interventions and endocrine therapy. Enhanced screening and surveillance.

Telehealth applications can be extended to tertiary prevention of breast cancer as well. Using a face-to-face videoconference strategy to supervise physical exercise in women undergoing primary treatment for breast cancer stages I-III (ABRACE) has been hypothesized to yield better physical and psychological outcomes compared with a health education program alone [36]. Similarly, wearable devices have been shown to significantly improve physical activity, weight control, and overall health in breast cancer survivors [37]. In the future, medical wearables with AI and advanced sensors may be able to detect early-stage breast cancer through noninvasive procedures. For example, a proof-of-concept ultrasonic breast patch (cUSBr-Patch) has been developed that can detect cysts as small as 0.03 cm in diameter, providing a noninvasive way to monitor real-time changes in breast tissue [38].

Another application of technology in breast cancer prevention is in identifying higher-risk individuals for recommendation for screening via risk prediction models (Table 6). Predominantly developed and validated within Caucasian populations, these models have shown modest discriminative power in validation studies with area under the curve values generally ranging between 0.7 and 0.8. The risk factors used are a composite of nonmodifiable elements—age, age at menarche, age at first live birth, additional birth history, family history, age at menopause, and mammogram outcomes—and modifiable elements, notably alcohol consumption and BMI, which provide avenues for risk reduction interventions.

**Table 6:** Breast cancer risk prediction models.

Model	Study design	Study size (cases/controls)	Remarks
Gail et al [39]	Nested case-control	2852/5998	Pioneering modeling study. Focus on reproductive factors.
Rosner and Colditz [40]	Cohort	2249/91,381	Inclusion of age at multiple birth and has been validated with the inclusion of modifiable risk factors.
Breast Cancer Surveillance Consortium; Kerlikowske et al [41]	Cohort	14,766/1,110,250	Inclusion of radiologic findings such as BI-RADS^a^.
Eriksson et al [42]	Case-control	433/2165	Emphasis on short-term risk prediction. Used computer-generated BI-RADS^a^.
Breast and Ovarian Analysis of Disease Incidence and Carrier Estimation Algorithm; Antoniou et al [43]	Case-control	1484/156 multiple-case families	Focus on mutation carrier (BRCA1/BRCA2) probabilities.

^a^BI-RADS: Breast Imaging Reporting and Data System.

The use of risk prediction techniques has enabled us to provide general advice on factors that can lower the risk of breast cancer and to recommend tailored mammography screening frequencies for each woman based on her individual risk level, as a primary prevention strategy. The polygenic risk score (PRS) has been particularly helpful in identifying individuals at a high risk of developing breast cancer. This allows for targeted interventions aimed at primary prevention, such as personalized lifestyle modifications, preventive therapies, and individual counseling.

In addition, PRS has enabled the stratification of individuals for enhanced screening and surveillance, thereby aiding in the early detection of breast cancer, which aligns with secondary prevention efforts. Furthermore, PRS has the potential to guide tertiary prevention strategies by identifying those who may benefit from more aggressive treatment or preventive measures, such as chemoprevention or risk-reducing surgery both before and after cancer diagnosis to decrease the advanced or recurrent cancer disease burden. Electronic breast cancer risk assessment tools are commonly used to estimate an individual’s risk of developing breast cancer. However, it is important to consider both genetic and environmental factors in order to accurately assess the absolute risk for each person in the population [44]. This comprehensive approach allows women to make informed decisions about lifestyle choices and preventive interventions based on their personal risk level and values [33]. Women with a family history of cancer and who are in good overall health should receive personalized care and risk management, while patients with cancer should be educated about how genetic germline mutations may impact their ongoing treatment, follow-up care, and secondary prevention [45]. With the advancement of patient-driven platforms such as HexaMed and DCGen, risk assessment is becoming more personalized and tailored to each individual.

Mobile apps are also used for prevention in breast cancer, especially among marginalized populations and women at increased risk. These apps have been proven effective in increasing mammography awareness and positive decision-making [46]. In addition, mobile apps for primary prevention aim to promote healthy behaviors that reduce the risk of breast cancer [47,48]. These apps primarily target women at high risk for breast cancer, such as postmenopausal women with high Gail risk scores [45] and *BRCA* mutation carriers [49,50]. Recently, studies such as the PREVENTION e-platform have been developed to provide personalized breast cancer information and support tailored to different risk levels [51].

Mobiles apps for tertiary prevention of breast cancer focus on clinical care coordination and health-related quality of life interventions [52]. Cancer care coordination focuses on support and communication between patients with breast cancer and physicians. Specific aspects of coordination include symptomology management, medication adherence, and ambulatory surgery. Health-related quality of life apps targeted general lifestyle management, weight management, depression, and breast cancer-related distress, social support, sleep, and physical activity during and after breast cancer diagnosis. Patients and survivors expressed a preference for receiving clinical care coordination support and quality-of-life interventions through mobile apps, indicating a shift away from standard practices [52].

### Case Study 2: Diabetes

Diabetes management, amidst its increasing global burden, presents challenges for both individual patients and the broader public health landscape [53]. The global surge in obesity and sedentary lifestyles has amplified the incidence of diabetes [54,55]. Meanwhile, our understanding of and diagnosis of the disease have advanced to recognize the need for earlier, more personalized care. This is reflected by the American Diabetes Association’s changing both the naming and the diagnostic criteria of various stages of hyperglycemia, including hemoglobin A_1c_ (HbA_1c_) as a diagnostic standard and accepting the concept of “prediabetes” (Table 7) [56,57]. However, traditional health care approaches have encountered significant limitations in preventing, diagnosing early, and mitigating complications associated with the disease. Owing to its gradual onset, lifestyle-related risk factors, and the absence of early symptoms, diabetes proves challenging to both prevent and detect in its initial stages. Moreover, its management is complicated by the necessity for frequent blood glucose monitoring, which yields only intermittent data on glucose levels. Furthermore, treatment regimens typically require strict adherence to medications that are prescribed based on generic guidelines. These guidelines do not accommodate individual patient differences, potentially compromising the effectiveness of the treatment [58].

**Table 7:** The American Diabetes Association evolution of diabetes as diagnosis.

Definitions	Diagnosis criteria	Year
IGT^a^	2-hour glucose levels 140-199 mg/dL	1979-1997
IFG^b^	Fasting glucose 110-125 mg/dL	1997, 1999
IFG	Fasting plasma glucose 100-125 mg/dL	2003-2006
Borderline HbA_1c_^c^	HbA_1c_^c^ 6.0%-5.7%	2009-2010
Prediabetes	IGT or IFG or borderline HbA_1c_^c^	2010

^a^IGT: impaired glucose tolerance.

^b^IFG: impaired fasting glucose.

^c^HbA_1c_: hemoglobin A_1c._

Digital health technology may be capable of improving how diabetes is prevented, screened, and managed. For one, digital health technology offers a promising avenue for primary prevention of diabetes by enabling personalized lifestyle and dietary interventions. Wearables and mobile apps are crucial for encouraging lifestyle changes, such as increasing physical activity and aiding in smoking cessation, by providing real-time feedback and monitoring. Furthermore, AI-driven personalized nutrition plans, tailored to an individual’s glycemic responses and microbiota profiles, play a vital role in optimizing metabolic health. Noom, for instance, is an app that provides personalized recommendations for nutrition, weight loss, and physical activity by improving user engagement and self-improvement [59,60]. Through these technologies, health care can adopt a more proactive and preventive stance, targeting key dietary and lifestyle risk factors effectively.

Digital health technology can also enhance secondary diabetes prevention by enabling noninvasive diagnosis and identifying individuals at high risk. Traditionally, the identification of individuals at high risk of diabetes depended on statistical models, some of which required fasting blood glucose tests (Table 8). These diabetes risk prediction models incorporate a mix of nonmodifiable risk factors, including age, family history of diabetes, comorbidities such as hypertension and cardiovascular disease, and laboratory test results, including blood glucose, lipids, and uric acid levels.

**Table 8:** Diabetes risk prediction models.

Model	Cohort name	Study size (cases/control)	Remarks
Lindström and Tuomilehto [61]	FINRISK	182/4253	Model based only on lifestyle factors.
Balkau et al [62]	DESIR^a^	203/3611	Genetic polymorphisms included as a risk factor.
Hippisley-Cox et al [63]	QResearch database	78,081/2,462,672	Recognized socioeconomic status as a risk factor.
Kahn et al [64]	ARIC^b^	1821/7766	Offers multiple models for varying levels of available clinical data.

^a^DESIR: Data from an Epidemiological Study on the Insulin Resistance Syndrome.

^b^ARIC: Atherosclerosis Risk in Communities.

More recently, modifiable risk factors such as alcohol consumption, smoking, physical activity, waist circumference, and BMI have been integrated, offering opportunities for risk reduction interventions. Unfortunately, traditional models have demonstrated modest discriminative ability, with area under the curve values typically ranging from 0.6 to 0.7. On the other hand, recent AI-developed models have shown themselves to be capable of predictive population risk stratification, allowing for targeted interventions and showing high sensitivity and specificity. One model, for example, was developed to predict prediabetes and type 2 diabetes based on 1415 Indians and reported 99.5% sensitivity and 99.07% accuracy [65]. This approach of using technology to identify individuals at high risk of diabetes streamlines resource allocation and improves prevention efforts, marking a shift toward proactive diabetes management.

Tertiary prevention of diabetes using digital health technology has shown the most progress in recent years. One example is continuous glucose monitoring (CGM), a wearable device that provides continuous, noninvasive monitoring of glucose levels and is capable of alerting patients to significant blood glucose swings. One such instance, Freestyle Libre, a CGM device, measures glucose levels in cellular fluids instead of serum glucose, offering real-time, noninvasive readings and hypo- and hyperglycemia alerts. The adoption of CGM devices has shown significant reductions in HbA_1c_, as well as improved participant-reported quality of life [66].

AI has also been used to work in conjunction with existing clinical practices, namely, facilitating the diagnosis of diabetic complications such as retinopathy and foot ulcers. For example, Lam et al [67] used a convoluted neural network to develop a system for identifying retinal lesion from ophthalmologic photographs, reporting 98% accuracy and a receiver operating characteristic curve of 99%. In another instance, Keel et al [68] reported 92% and 94% sensitivity and specificity, respectively, in identifying diabetic retinopathy in outpatient conditions. Siren Care’s Siren Socks is a particularly creative approach to managing diabetic ulcers, using socks to measure foot temperature of patients with diabetes to detect diabetic foot neuropathy, ulcer, and injury [69].

## Key Considerations for Digital Health Technology Applications

Despite its potential benefits, the application of modern digital health technology in preventive medicine must proceed with caution. For one, since access to digital devices, the web, and digital literacy depend on socioeconomic factors, adopting technology to provide preventive medicine services may further exacerbate existing health disparities. Services such as mobile apps for cancer prevention or diabetes management, despite their potential, will be of limited use if they are not accessible or effectively promoted to marginalized groups. Conversely, digital health technology has the potential to contribute to health equity when used correctly. Telemedicine, for example, significantly enhances health care access for remote populations, individuals with disabilities, and low-income groups by reducing travel costs and barriers to care. To provide digital health care where it is needed, it is important to advocate for the development of communications and technology infrastructure in marginalized communities and support policies that promote inclusive medical practice [70,71].

Another point of concern is the potential challenges posed by an overreliance on digital devices. Excessive use of electronic devices has been associated with both physical and mental health hazards, such as computer vision syndrome, obesity, and developmental disorders [72-74]. Overuse of digital health devices may cause harm instead of promoting health. There is also the risk of overreliance on electronic systems for health care decisions and delivery, which could detract from the judgment of experienced health care providers. Despite developments in AI and self-health management mobile apps, these electronic devices must be recognized as tools to aid, not replace, the therapeutic efforts of patients and health care providers.

Ethical concerns also warrant attention. The integration of genomics and personal data into health care platforms raises concerns, especially due to possible discrimination. Although genetic data can aid in prevention, there have been concerns that they could be used to deny insurance or employment to individuals or their families [75], as has materialized in some legal cases [76]. Unauthorized access to and misuse of health records could also lead to discrimination and breaches of confidentiality, ultimately undermining trust in digital health systems. There is already a robust body of literature addressing concerns about patient data security [77-79]. Future and continuous efforts must be focused on establishing robust safeguards and ethical guidelines to protect against such discrimination, breaches, and misuse, as well as on policy to ensure that the advancement of digital health technologies does not compromise health care ethics.

Finally, as much as the field of digital technology and its applications in preventive medicine are exploding, there is a critical need for rigorous testing of safety, efficacy, and data validation. One study found that most digital health startups (44%) scored zero, the lowest score, in clinical robustness as measured by regulatory filings and clinical trials [80]. To move past being a mere buzz and truly become future health care, digital health technology requires in-depth, longitudinal studies and user experience research.

This review provides a comprehensive, forward-looking perspective on the future of preventive medicine. By examining current trends, technological advancements, and case studies, this study offers valuable insights into how preventive health care is evolving in the digital era toward a more proactive, personalized, and patient-centered approach to health care. Furthermore, this study identifies the potential of digital health technologies to transform preventive medicine, providing concrete examples of how digital tools are being leveraged for primary, secondary, and tertiary prevention and highlighting the need to address disparities in technology access, prevent overreliance on digital tools, safeguard patient data, and ensure clinical validity of digital health applications.

Despite these strengths, this viewpoint is based largely on observations and trends within the field rather than empirical research. As such, while it is an expert opinion familiar with industry trends and the applications of technology in preventive medicine, this perspective may not fully capture all dimensions of digital health application in prevention. Despite this limitation, we have sought to avoid potential biases by drawing on diverse sources to provide a balanced assessment of the role of digital health in preventive medicine.

Given the rapid advances in digital health technology and its potential to transform preventive medicine, further investigation is warranted. This exploration must focus not only on improving technological capabilities but also on addressing concerns related to health equity, responsible usage, ethics, and clinical validation that accompany such advancements. Only then can we better guarantee that these innovations deliver safe, effective, and equitable health outcomes for all sections of society.

## Summary

The future trend of health care is expected to be that of a rise in the older adult population, a shift from cure to prevention, increased use of digital health technology and telemedicine, and a gradual left shift of diseases and the focus of health care. In this review, the concept of primary, secondary, and tertiary prevention in the scope of digital health care was highlighted, as well as breast cancer and diabetes prevention as case studies of the actual application of digital technology in the realm of health care.

The role of preventive medicine is changing as new medical and digital technologies are developed and integrated with health care. These advancements have expanded the reach of preventive services, allowed for remote monitoring, and enabled personalized health interventions, leading to a more patient-centric and data-driven approach to prevention and health care. As a result, health care is closer, faster, and delivered at a lower cost to patients, while patients are empowered with personalized health records and improved patient engagement.

In this study, we have seen that the integration of digital technologies into preventive medicine marks a pivotal evolution in health care. The pace at which technology is evolving is unprecedented. It should be noted, however, that the practical application of these innovations requires robust research to validate the efficacy and safety of digital health technologies in preventive medicine, ensuring that future health care systems are both innovative and inclusive, as well as rigorous validation through large-scale population studies. Looking ahead, we anticipate a transformative health care landscape where personalized preventive measures become fundamental, supported by evidence of efficacy and applicability of digital technologies in preventive medicine.

## Abbreviations

AI: artificial intelligence
ASD: autism spectrum disorder
CGM: continuous glucose monitoring
DSM: Diagnostic and Statistical Manual of Mental Disorders
DSM-III: Diagnostic and Statistical Manual of Mental Disorders (Third Edition)
DSM-IV-TR: Diagnostic and Statistical Manual of Mental Disorders (Fourth Edition, Text Revision)
DSM-V: Diagnostic and Statistical Manual of Mental Disorders (Fifth Edition)
HbA1c: hemoglobin A1c
ML: machine learning
PRS: polygenic risk score
